# Multiple Ulcers in the Ileum and Lymphadenopathy Following the Usage of Methotrexate in a Patient With Rheumatoid Arthritis: A Case Report

**DOI:** 10.7759/cureus.53406

**Published:** 2024-02-01

**Authors:** Ryuichi Ohta, Tsuyoshi Mishiro, Yuta Horinishi, Chiaki Sano

**Affiliations:** 1 Community Care, Unnan City Hospital, Unnan, JPN; 2 Internal Medicine, Unnan City Hospital, Unnan, JPN; 3 Community Medicine Management, Faculty of Medicine, Shimane University, Izumo, JPN

**Keywords:** rural, family medicine, general medicine, gastrointestinal hemorrhage, lymphadenopathy, ileal ulcers, severe anemia, elderly onset rheumatoid arthritis, methotrexate-induced complications

## Abstract

This case report aims to highlight a rare occurrence of severe anemia and lymphadenopathy secondary to methotrexate (MTX)-induced ileal mucosa damage in a patient with elderly onset rheumatoid arthritis (EORA). We present the case of a 72-year-old female with a history of EORA, treated with MTX, who exhibited hematochezia without accompanying pain, diarrhea, or known infectious contacts. Diagnostic investigations included imaging and endoscopic procedures. The patient's presentation of severe anemia was atypical, given the absence of significant pain or discomfort associated with EORA. The lack of active bleeding observed during endoscopy, coupled with multiple ileal ulcers, suggested a chronic progression of mucosal damage. Laboratory findings, including normal lactate dehydrogenase, soluble interleukin-2 receptor levels, and the absence of malignancy in mucosal biopsies, ruled out MTX-induced lymphoma. The patient's condition improved with the cessation of MTX and the introduction of symptomatic treatment and anemia management. This case underscores the need for vigilant monitoring and comprehensive evaluation in patients with RA, especially the elderly, treated with MTX. It also highlights the importance of considering drug-induced complications in the differential diagnosis of anemia. The case demonstrates the necessity of a personalized approach to treatment, emphasizing regular follow-ups and adjustments based on the patient's response to therapy. This report contributes to the growing body of evidence on the complexities of managing RA in the elderly, particularly regarding the side effects of common medications like MTX.

## Introduction

Rheumatoid arthritis (RA), a chronic inflammatory condition primarily impacting the joints, manifests uniquely in older adults as elderly onset rheumatoid arthritis (EORA) [1]. Within the treatment paradigm for EORA, methotrexate (MTX) has risen as a fundamental treatment, instrumental in reducing disease activity and steering patients toward remission [2]. However, MTX administration, especially in elderly patients, presents distinct challenges [3]. The heightened susceptibility of senior individuals with EORA demands prudent management due to the array of potential adverse effects associated with MTX [3].

In managing EORA with MTX, stringent monitoring of its side effects is essential, requiring detailed medical oversight and immediate intervention. This necessity is accentuated by the variability in both the side effects of MTX and the manifestation of EORA symptoms, which can vary from minor discomforts to severe, potentially life-endangering conditions [4]. Gastrointestinal issues are particularly common among these complications [5]. In certain instances, these symptoms may progress to critical states such as gastrointestinal bleeding and profound anemia, thereby significantly endangering the patient's health and safety [6,7].

This case report presents a 72-year-old female diagnosed with EORA and undergoing MTX treatment, who developed acute gastrointestinal bleeding, severe anemia, and abdominal lymphadenopathy. A comprehensive medical examination revealed multiple ileal ulcers, a consequence attributed to MTX-induced inflammation of the stomach lining, concomitant with reactive lymphadenopathy. This case illustrates the intricate challenges in diagnosing and treating acute anemia due to gastrointestinal bleeding in patients with EORA, further complicated by MTX use. The lessons from this case emphasize the imperative for increased vigilance and diagnostic precision in treating older RA patients, particularly when utilizing medications like MTX that have a high risk of serious side effects.

## Case presentation

A 72-year-old woman presented at a rural community hospital with the primary symptom of hematochezia. The day before admission, she experienced nausea and abdominal bloating. On the morning of admission day, she had multiple episodes of hematochezia. She reported no chest, abdominal, or back pain, or diarrhea. There were no sick contacts or people with similar symptoms around her. She had no recent travel or unusual dietary history that might suggest infection. Her medical history included four years of EORA. She was on 16 mg/week of MTX and 5 mg/week of foliate. An upper endoscopy and fecal occult blood test performed three years prior showed no abnormalities.

At the time of the visit, her vital signs were as follows: blood pressure 157/88 mmHg, pulse rate 95 beats/min, body temperature 36.8 °C, respiratory rate 21 breaths/min, and oxygen saturation 98% on room air. She was oriented to time, place, and person. The physical examination revealed pale conjunctiva without jaundice and mild abdominal tenderness without rebound or percussion tenderness. No other neurological abnormalities, chest irregularities, or skin eruptions were observed. Laboratory tests indicated severe anemia but no signs of inflammation, as her hemoglobin level two months ago was 14.5 g/dL (Table 1).

**Table 1:** Initial laboratory data of the patient. MCV: mean corpuscular volume; eGFR: estimated glomerular filtration rate; CK: creatine kinase; CRP: C-reactive protein.

Parameter	Level	Reference
White blood cells	12.7	3.5–9.1 × 10^3^/μL
Neutrophils	75.7	44.0–72.0%
Lymphocytes	19.8	18.0–59.0%
Monocytes	3.4	0.0–12.0%
Eosinophils	0.5	0.0–10.0%
Basophils	0.6	0.0–3.0%
Red blood cells	2.96	3.76–5.50 × 10^6^/μL
Hemoglobin	8.9	11.3–15.2 g/dL
Hematocrit	26.3	33.4–44.9%
MCV	88.9	79.0–100.0 fL
Platelets	32.4	13.0–36.9 × 10^4^/μL
Total protein	6.3	6.5–8.3 g/dL
Albumin	3.8	3.8–5.3 g/dL
Total bilirubin	0.4	0.2–1.2 mg/dL
Aspartate aminotransferase	34	8–38 IU/L
Alanine aminotransferase	45	4–43 IU/L
Alkaline phosphatase	70	106–322 U/L
γ-Glutamyl transpeptidase	21	<48 IU/L
Lactate dehydrogenase	203	121–245 U/L
Blood urea nitrogen	34.6	8–20 mg/dL
Creatinine	0.71	0.40–1.10 mg/dL
eGFR	61.3	>60.0 mL/min/L
Serum Na	138	135–150 mEq/L
Serum K	4.4	3.5–5.3 mEq/L
Serum Cl	103	98–110 mEq/L
Serum Ca	9.1	8.8–10.2 mg/dL
Serum P	3.2	2.7–4.6 mg/dL
Serum Mg	1.9	1.8–2.3 mg/dL
Serum iron	42	43-172 mg/dL
Ferritin	51.3	14.4–303.7 ng/mL
CK	64	56–244 U/L
CRP	0.04	<0.30 mg/dL

**Figure 1:**
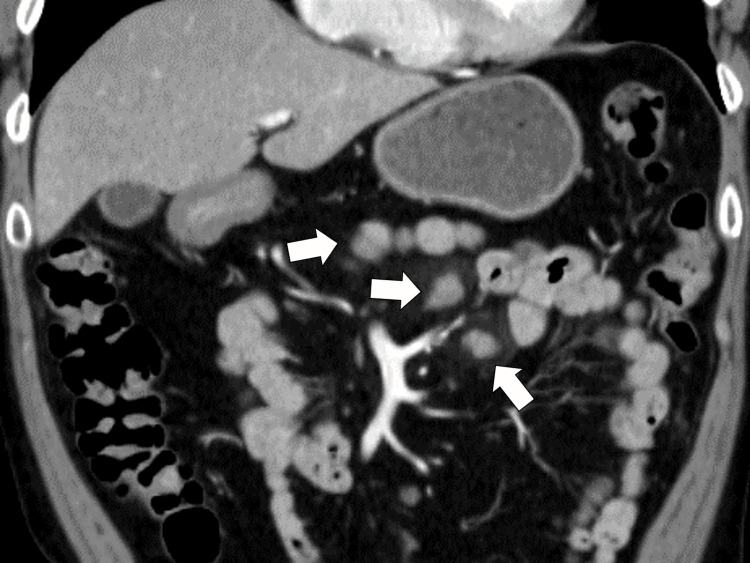
Abdominal computed tomography with contrast clarifying local lymphadenopathy around the stomach without any extravasation on the stomach's mucus (white arrows).

Abdominal CT with contrast showed local lymphadenopathy around the stomach without extravasation in the gastric mucosa and without continual bleeding (Figure 1).

**Figure 2:**
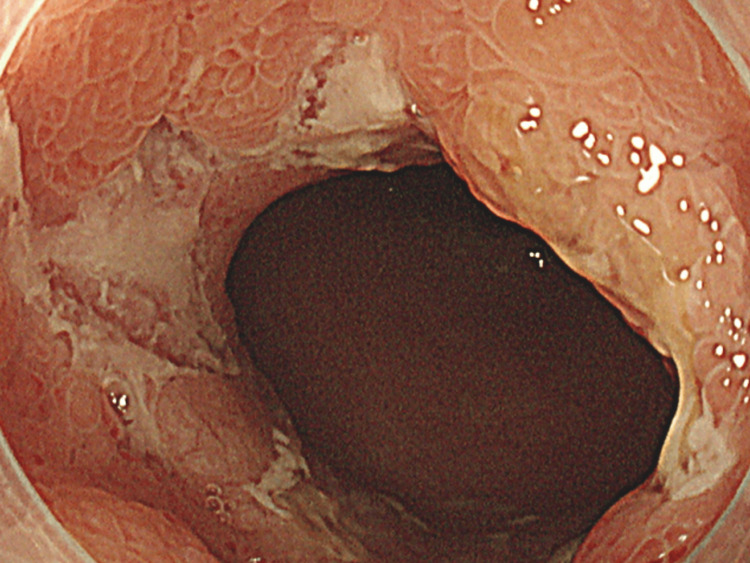
Lower gastrointestinal endoscopy clarifying multiple ulcers on the mucosa of the ileum.

An urgent lower gastrointestinal endoscopy identified multiple ulcers on the mucosa of the ileum (Figure 2). 

Subsequently, an urgent upper gastroduodenal endoscopy revealed no bleeding. Based on these findings, the patient was diagnosed with MTX-induced ileal ulcer and suspected MTX-induced lymphoma. Additional tests showed normal lactate dehydrogenase and soluble interleukin-2 receptors. Biopsies from the stomach, duodenum, ileum, and colon mucosa revealed no malignancy indicative of lymphoma. The patient was treated with oral vonoprazan (20 mg) and rebamipide (300 mg) per day, and MTX was discontinued. One week later, her anemia improved to a hemoglobin level of 10 g/dL, and she experienced no nausea or vomiting. She was discharged with a full return to daily activities and continued follow-up in the outpatient department without anemia recurrence.

## Discussion

This case report details a unique presentation of severe anemia resulting from MTX-related damage to the ileal mucosa, accompanied by lymphadenopathy. While gastrointestinal complications are commonly associated with MTX treatment, instances leading to critical conditions are rare. Given the frequent use of MTX in RA management, it is crucial for clinicians to thoroughly investigate progressive anemia in RA patients, considering potential MTX-related complications affecting the gastrointestinal mucosa.

The diverse nature of MTX complications necessitates that physicians, particularly in rural areas, meticulously monitor their patients on MTX for signs of progressive anemia [8,9]. In this case, a 72-year-old female diagnosed with EORA exhibited hematochezia, notably without pain, diarrhea, or known infectious contacts, highlighting the need for increased attention to atypical symptom presentations. This case emphasizes the importance of comprehensive evaluation in RA patients receiving MTX treatment, especially when they present with non-specific symptoms such as nausea and vomiting. Patients with RA on MTX therapy may experience mild anemia due to suboptimal disease control or disease flares [10,11]. It is essential to distinguish between anemia caused by chronic inflammation and that resulting from gastrointestinal bleeding. The absence of inflammatory indicators, such as elevated CRP, as demonstrated in this case, indicates the necessity for further investigation into potential causes of gastrointestinal bleeding [12].

The approach to investigating anemia in MTX-treated patients should be thorough. This case revealed severe anemia, a surprising finding given the lack of significant pain or discomfort typically associated with EORA. A diagnostic approach combining both imaging and endoscopy was instrumental in determining the extent of gastrointestinal involvement [13]. The lack of active bleeding observed during endoscopy, alongside the discovery of multiple ulcers in the ileal mucosa, pointed to progressive mucosal damage rather than acute gastrointestinal bleeding [14,15]. The absence of previous anemia episodes and normal findings from earlier upper and lower endoscopies had delayed more immediate gastroduodenal exploration. Additionally, MTX can cause an increase in the mean corpuscular volume (MCV) of red blood cells [16]. Detecting changes in MCV is crucial, as iron deficiency anemia from bleeding typically leads to a decrease in MCV [16]. Therefore, evaluating anemia in patients with EORA on MTX should include an assessment of inflammation and regular checks for gastrointestinal tract damage through endoscopy, rather than solely relying on laboratory data.

Another significant aspect of this case is the differential diagnosis of lymphadenopathy in patients on MTX, including MTX-induced lymphoma, a rare but notable long-term complication of MTX therapy that necessitates its discontinuation [17]. This patient's normal lactate dehydrogenase and soluble interleukin-2 receptor levels, combined with the absence of malignancy in mucosal biopsies, excluded lymphoma. The management strategy involved immediate cessation of MTX, providing symptomatic relief and addressing the anemia [18]. The patient's positive response to treatment, including anemia resolution and overall health improvement, further confirms MTX's role in her condition.

## Conclusions

This case illuminates several key clinical insights. It underscores the importance of ongoing monitoring and reassessment of patients undergoing long-term MTX therapy, especially when new or escalating gastrointestinal symptoms arise. It also highlights the necessity of considering drug-induced complications in the differential diagnosis of anemia in RA patients. Lastly, it advocates for personalized treatment plans tailored to each patient's specific presentation and therapeutic response.
